# Exploring the Relationship between Urban Street Spatial Patterns and Street Vitality: A Case Study of Guiyang, China

**DOI:** 10.3390/ijerph20021646

**Published:** 2023-01-16

**Authors:** Junyue Yang, Xiaomei Li, Jia Du, Canhui Cheng

**Affiliations:** 1 College of Architecture and Urban Planning, Guizhou University, Guiyang 550025, China; 2 School of Architecture and Urban Planning, Chongqing University, Chongqing 400044, China

**Keywords:** street spatial patterns, street vitality, street design, big data, China

## Abstract

Understanding how street spatial patterns are related to street vitality is conducive to enhancing effective urban and street design. Such analysis is facilitated by big data technology as it enables more accurate methods. This study cites data from street view imagery (SVI) and points of interest (POI) to assess street vitality strength after the classification of street spatial and vitality types to explore the relationship between street spatial patterns and street vitality with a further discussion on the layout features of street vitality and its strength in various street spatial patterns. First, street spatial patterns are quantified based on SVI, which are further classified using principal component analysis and cluster analysis; POI data are then introduced to identify street vitality patterns and layout, and the strength of street vitality is evaluated using spatial overlay analysis. Finally, relevance analysis is explored to cast light on the relationship between street vitality layout and street spatial patterns by overlaying street spatial pattern, street vitality types, and street vitality strength in the grid cells. This paper takes the urban area of Guiyang, China, as an example and the analysis shows that a pattern is discovered in Guiyang regarding the layout of street vitality types and vitality strengths across different street spatial patterns; compact street spaces should be prioritized in designing street space renovation; and cultural leisure vitality is most adaptive to street spatial patterns. Based on big data and using grids to understand the intrinsic relationship between street spatial patterns and the type and strength of street vitality, this paper brings more options to urban street studies in terms of perspective and methodology.

## 1. Introduction

Streets are the main spaces of public life in cities, carrying diversity and vitality [1]. At the same time, streets help to shape a city by determining the way people move and stay [2]. Peoples’ impressions of the urban space are directly impacted by the spatial patterns of streets. In addition, a large number of scholars believe that mixed street vitality and high street vitality strength are more conducive to sustainable development [3,4]. However, the spatial forms of streets in urban areas are different, and the distribution and development trends of streets with different space morphology are different [5]; notably, the very different space morphology of urban streets has different influences on the urban, social, economic, cultural, and natural environment [6]. Streets with different spatial forms provide people with different ways of living and activities [7,8,9]. Remarkably, mixed land use is one of the characteristics of urban street spaces [10], which means that the urban street space contains rich urban functions and multiple urban vitality, and the vitality of a street is closely related to the physical space form of the street [11].

In this context, a large number of studies on the vitality and spatial patterns of urban streets have emerged, but the assessment of street vitality and the classification of street spatial patterns are largely based on manual survey methods, such as questionnaires, field statistics, and measurements [12,13,14]. In recent years, new perspectives emerging from big-data-based computer analytics and geographic information science and technology are facilitating space analysis of urban streets [15,16,17]. Compared with the traditional manual collection of information and data, urban big data that promise fast and free-of-charge access show a huge advantage in that big data do not only carry objective physical information but also preferences and characteristics regarding peoples’ activities, making it highly applicable and effective in the analysis of the vitality and spatial patterns of urban streets. Many urban street studies have already been using multiple sources of big data such as street view imagery (SVI) and points of interest (POI).

Existing studies of street vitality are currently dominated by vitality evaluation. Liu et al., used POI data as the basis for urban vitality identification, which was combined with the features of land use in surrounding areas to calculate the space–time layout of vitality and analyze the influencing factors of layout evolution [18]. Zeng et al. used big data to create spatially explicit indices to identify spatial patterns of vitality in cities and developed a graded assessment of vitality [19]. Chen et al. cited POI and Weibo check-in geographic markers to analyze vitality distribution and indices [20]. These attempts to study urban vitality have demonstrated the feasibility of analytical methods that use big data to reflect the characteristics of population activity as a means to obtain an accurate distribution of urban vitality [21]. They equally prove the validity of geo-information data in the analysis of the spatial pattern and vitality of streets [22]. However, current studies on urban vitality tend to neglect the interaction of vitality and built-up environment [23] and few have provided a categorization framework for vitality understanding, which limits the creative value of vitality analysis for urban design [24].

Street space morphology studies, on the one hand, evaluate the quality of street space by analyzing peoples’ perceptions of street space from the public viewpoint [25,26]. Wu et al. formed an SVI-based random forest model trained on perceptual samples to assess the visual quality of street space [27]. Hu et al. accessed POI data to identify street functions, which were further integrated into an SVI semantic segmentation analysis of street space to illustrate the impacts of function variance on street spatial patterns [28]. On the other hand, many studies focus on the relationship between such patterns and the speed of the vehicles passing through [29] them, as the street patterns change drivers’ perceptions of street length and the density of buildings and consequently, their speed [30]. The influencing variables on street patterns, in turn, include urban traffic volumes and traffic demand [31]. Differences in spatial patterns of the streets and their design speed also influence their vitality [32]. Evidence also shows that street spatial patterns have implications for urban microclimate and pollution, most of which are SVI-based analyses [33,34,35]. In general, SVI is an important source of data for street space studies [36], and the mutual relations of street spatial patterns and vitality are supported by a wide range of studies.

There also exist many studies which act as support to the analysis of street vitality and spatial patterns, while we can find equally abundant evidence for the relationship between the two. Studies on how the environmental features of buildings improve street vitality can be dated back to the 1960s [37]. Xu et al. proposed a framework to assess street vitality, which includes street pattern, street business type, and street accessibility [38]. Long et al. discovered a positive correlation between street function mix and its ‘walk score’ [39]. Jiang et al. based the analysis of a human-scale investigation of vitality vis à vis environmental features of buildings on the various types of LBS (location-based service) data, such as bus cards, taxis, mobile phones, and taxi trajectory [40]. Xia et al. studied city vitality in daytime and at night with the help of nighttime lighting data and extended the analysis to the spatial relationship between land use intensity and urban vitality [41].

This study emphasizes the interactive relationship between the spatial patterns of streets, vitality types, and vitality strength (the more vitality types in the same area, the higher the vitality strength). It explores the differences in street spatial patterns, which are believed to be a perspective to the spatial layout features of street vitality types and strength, so as to contribute to enriching the methodology and angles towards urban street space renovation design and the shaping of street spatial vitality.

Street spatial patterns embody the feature of the area by integrating the function of the buildings and the morphological elements. As such, streets and buildings, the functions the buildings have, and the vitality of such functions are closely interconnected [42]. Streets are the core space for public life in cities and should highlight the integration of street spatial patterns and the vitality that takes the function of the street as the foundation [43]. For a long time, planners and designers have been working to create streets with a unique identity and high vitality [44], which can only be accomplished after understanding the interactions between the spatial pattern, vitality type, and strength of streets, allowing the full release of the potential in street spatial patterns and vitality during the urban and street design process [45]. Hence, the main concerns of this study are:

- Exploring the classification of urban street spatial patterns;
- Exploring methods for determining street vitality types and vitality strength;
- Analyzing the inter-relationship between the spatial pattern, vitality type, and vitality strength of streets.

This paper proposes a classification method of street spaces and an evaluation method of street vitality based on multi-sourced data based on ArcGIS. Such methodology and quests into a perspective on urban street analysis that integrates street spatial pattern and vitality serve as compliments to the existing system of urban street analysis and are more suitable for the urban design of intensive city centers.

## 2. Research Scope and Data Processing

### 2.1. Research Scope

The scope of this paper is the city center of Guiyang, Guizhou, China. To be more specific, it is north of Beijing Road, south of the southern section of the central ring road; west of Huaxi Avenue, and east of Guiyang Ring Highway. The urban streets in the abovementioned scope are considered research subjects, which are cut by intersections. The total number of streets concerned in this study is 1153.

Guiyang is located in the mountain area of southwest China, and cities of its kind typically lack flat lands [46]. As a result of changing landscapes, streets in central Guiyang show high density and complex spatial patterns [47]. Moreover, as a city with a long history, Guiyang has many buildings and areas of historical importance. Our team has calculated the number of historical and cultural sites protected at the provincial- and municipal-level and the result is 39 in the study area alone. For example, the Qianling Mountain monuments and stone inscriptions (4,260,000 square meters), the ancient buildings of Guizhou Normal University (694,667 square meters) and the former residence of Mao Guangxiang and the site of warehouses for Forbidden City relics (404,290 square meters) are the three main sites. There are also seven more sites with areas larger than 10,000 square meters and seventeen larger than 1000 square meters. Among the 39 sites in the research area, 25 of them are adjacent to streets, meaning that these law-protected sites are key driving forces behind the changes in street spatial patterns in central Guiyang. Although facing huge demand due to a large population, the central area of the city has maintained diversity in spatial patterns. At the same time, as urbanization speeds up, the street functions in the central area continue to be more integrated and denser, resulting in a highly diverse combination of street function and spatial pattern. Therefore, the central area of Guiyang is an ideal research scope for the understanding of the relationship between the spatial pattern, vitality type, and vitality strength of urban streets.

### 2.2. Data Processing

**Figure 1:**
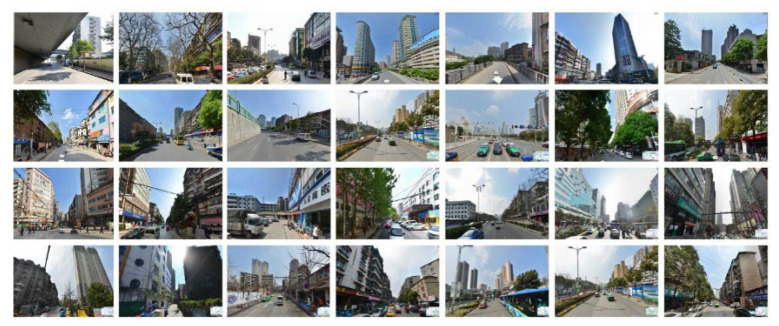
Tencent Streetview pictures of downtown Guiyang (selected).

A total of 1153 central points, latitude, and longitude coordinates of all streets in the research scope were collected. The starting point, ending point, and central points of street sections were used as anchors to crawl SVI data in Tencent Map, which resulted in three street photos for each section. A total of 3459 Tencent Streetview pictures of central Guiyang were collected, as shown in Figure 1. The OpenStreetMap (OSM) and satellite map of Guiyang were imported into ArcGIS to sketch all 1153 street sections in the research scope. The street centerlines were simplified to generate the street network in ArcGIS. In addition, POI data of the research scope were gathered using online tools, resulting in 95,522 entries of 11 categories.

## 3. Research Methodology

### 3.1. Street Space Classification 

#### 3.1.1. Establishing Street Space Indicators

**Figure 2:**
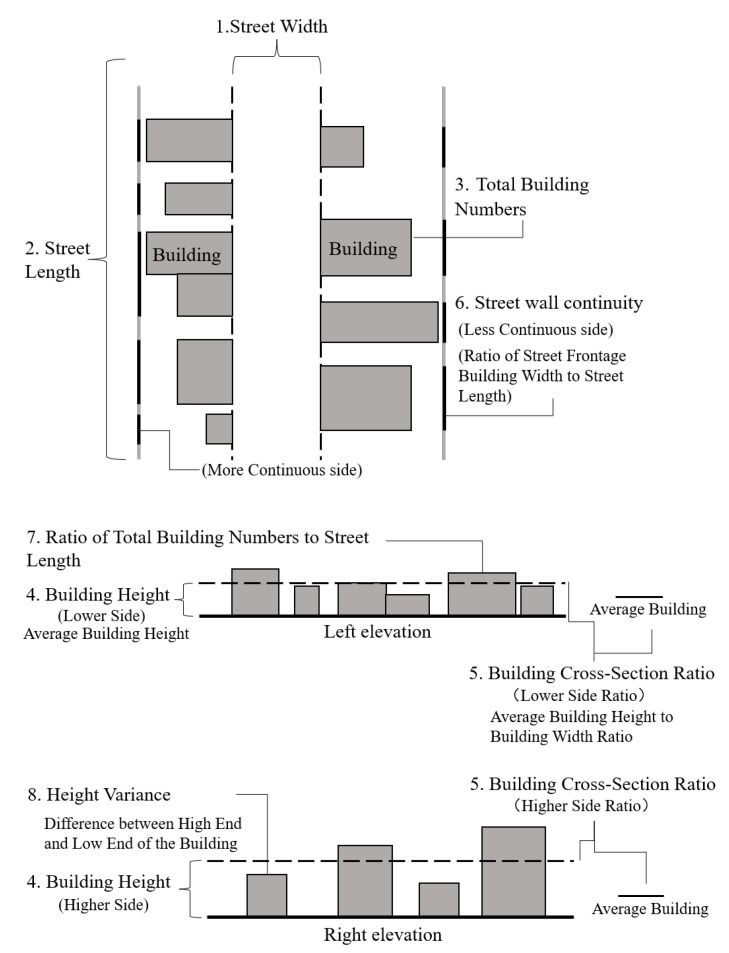
Quantitative street space indicator interpretation map [48].

From the perspective of humans’ perceptions of the enclosing state of street space, this paper draws on the street skeleton variable system proposed by Harvey Chester Wollaeger in 2014 [48] to include indicators which are street width, street length, total building numbers, building height, building cross-section ratio, street wall continuity, ratio of total building numbers to street length, and height variance. The definition and importance of each indicator is introduced in Table 1. The research classifies street space types by evaluating the enclosing state of street space. In view of the difference between the two sides of the street, 8 indicators are decomposed into 11 sub-indicators in Table 2. The detailed explanation of each spatial index is shown in Figure 2.

#### 3.1.2. Collection of Street Space Data Set

As shown in the indicator table, this study requires six data sets, namely, street length, street width, higher building height, lower building height, building width, and the number of buildings. Street length is calculated using the Calculate Geometry tool in ArcGIS and street width is calculated based on high-resolution satellite images.

As for building heights, widths, and numbers, four undergraduates with urban and rural planning and design backgrounds were recruited to make estimations based on the street view pictures and input into the software. They started with the collective measurement using a shared method to collect street building data from street view pictures and then worked individually to import data into ArcGIS street network attributes table. The imported data were converted into indicator data using the aggregated statistics tool in ArcGIS. In total, this took six days. Specifically, every undergraduate was required to complete 48–49 streets each day, and the import time of each street was controlled within 10 min; hence, each student was supposed to work less than 8 h a day.

The calculation of the mean and standard deviation for each indicator data set was completed as shown in Table 3.

A total of 7398 buildings were counted in the research scope. Most of the streets were two lanes in both directions in terms of average width. The mean heights of buildings fell in the range of 10.32–19.7, and most of the buildings had one floor or a couple of floors. Street wall continuity, which in general was high at 59% in dense places and 42% in sparse places, reflects the proportion of the length of the street versus building width.

This was based on the deviation calculation formula:

$$\mathrm{CV}=\frac{\mathrm{SD}}{\mathrm{Mean}}\times 100\%$$

In the formula, CV is the deviation value, SD is the standard deviation, and Mean is the mean. The CV values for each indicator were calculated (within a reasonable range of 5–35%) and it was found that the CV values for the street space indicators within the data collection area were all higher than the maximum skewness value of 35%, indicating that the street spaces in downtown Guiyang are significantly different with a wide range of street types.

#### 3.1.3. Street Spatial Pattern Identification

1. Principal Component Analysis (PCA)

PCA is conducted in SPSS on street space data, yielding a matrix as shown in Table 4. The criteria for the spatial classification of streets can be derived from the proportion of the components corresponding to the indicators. The data obtained were divided into four principal components with a total contribution of 64.86%, cumulatively.

In the component matrix, we selected data with component indicators greater than 0.6. In the first principal component, the indicators that met the criteria were building height (lower and higher) and street wall continuity (1.2); in the second principal component, it was the number of buildings; in the third and fourth principal components, there were no values that met the selection criteria. Hence, the three categories of building height, street wall continuity, and the number of buildings were used as the basis for classifying the street space for analysis in the next step.

2. Cluster Analysis

Building height, street wall continuity, and the number of buildings were used as the basis for cluster analysis of street spaces, which was conducted using Ward’s method (sum of squares of deviations). The indicators were grouped into four categories according to the results. As breakdown data show in Table 5, they corresponded to the four types of street space: closed, compact, semi-open, and open.

- Closed streets are characterized by high continuity of street walls and multi-floor or high buildings. The street space looks narrow from the human eye’s perspective;
- Compact streets are similar to closed streets in street wall continuity and building numbers, but most buildings in this type of street are ground-floor buildings or podiums of high-rise buildings. The buildings are lower than those in closed streets;
- Semi-open streets are occupied by fewer buildings and characterized by large gaps in the continuity of the street walls on both sides of the street, with large building spacing and independent distribution;
- Open streets are spacious, with squares, public buildings, or vacant lots appearing on both sides of the street. The wall continuity is low, and the obstruction of views is fewer.

### 3.2. Street Vitality Identification

#### 3.2.1. Street Vitality Categories

Urban street vitality is represented by the categories and frequency density of POI data. Based on the street type classification method of Better Street Plan in San Francisco [49] and the urban space characteristics of Guiyang city, this paper divides street vitality into four categories based on Baidu POI data categories, namely, residential, commercial, cultural leisure, and management and medicine (see Table 6). The residence category includes the buildings for people to live in and services, such as housekeeping, moving companies, wedding, elderly care, auto repair, renovation, car wash, and parking, among others; the commercial category includes facilities for personal, enterprise, and government consumption, such as enterprises, financial services, office buildings, accommodation, shopping, and catering services; cultural leisure refers to those facilities for entertainment, culture, and learning, among other purposes, such as tourism attractions, historical sites, education centers, research buildings, sports centers, and art performance buildings; management and medicine refer to the facilities required for the operation of the city and for the health care of residents, such as government buildings, municipal services, welfare centers, hospitals, clinics, and pharmacies.

#### 3.2.2. Street Vitality Categories and Levels 

This paper draws upon the San Francisco street type classification method and quantitative identification method of urban functional area [49]; the research divides the research area into grids of 500 m × 500 m. POI data are then cited to determine the vitality type of each grid, which is quoted as the vitality type of the streets in the grid [50]. This paper uses the 500 m × 500 m scale because the target area includes many historical sites, which occupy large areas. Furthermore, the local residence facilities are largely big, closed communities. Consequently, the street spaces that are the target of design and renovation scatter in such large objects. The 500 m × 500 m scale allows the grids to include street spaces that are possible for renovation and design, and is fit for maintaining a complete street spatial experience. Normally, 500 m is the distance for a 10–30 min walk, allowing pedestrians to have a more comfortable and complete street spatial experience and that of the street vitality.

Firstly, the frequency density of every POI category was calculated according to the following formula:

$$f_{i}=\frac{n_{i}}{\sum_{i=1}^{4}n_{i}}\left(i=1,2,3,4\right)$$

In the formula, $f_{i}$ denotes the frequency density of type i POI in the grid and $n_{i}$ is the number of type i POI in the grid.

Then, the frequency density of each POI category within each grid was compared to the total frequency density to determine the dominant POI category and thus the type of street vitality within that grid. The formula to calculate total frequency density of POI is as follows:

$$F_{i}=\frac{N_{i}}{N}\left(i=1,2,3,4\right)$$

In the formula, $F_{i}$ denotes the total frequency density of type i POI; $N_{i}$ is the number of type i POI; and N represents the number of all POI.

By overlaying the four vitality types, more vitality types appear in a grid, the higher the vitality strength of the grid that is considered, and the grid vitality strength is graded according to this method. Hence, streets can be classified into four levels: grids with four vitality types are high vitality grids, which correspond to high vitality streets; grids with three vitality types are relatively high vitality grids, which correspond to relatively high vitality streets; grids with two or one vitality type(s) are relatively low and low vitality grids, which correspond to relatively low and low vitality streets.

This paper overlays the maps of vitality grids with street spatial pattern layouts to calculate the proportion of different patterns in each grid. The pattern with the highest proportion becomes the pattern of the grid. As a result, the layout of street vitality types is generated by counting the number of grids with a certain type of street vitality under a certain spatial pattern. Then, the vitality strength level grids are overlaid with the grids of street spatial patterns to show the numbers of grids with a certain type of vitality under a certain spatial pattern, so as to generate the vitality strength layout of various spatial patterns.

## 4. Research Conclusion

### 4.1. Street Spatial Pattern Classification 

**Figure 3:**
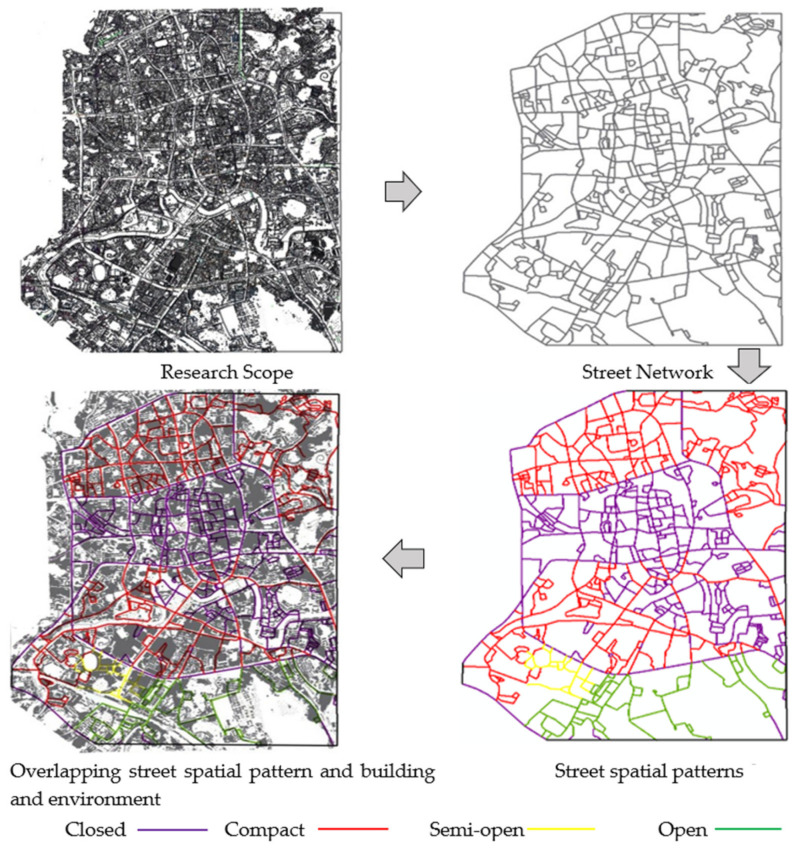
Process of street space classification.

The four street types are connected in the ArcGIS street network map according to the classification of indicator data to form a visual analysis model to obtain a spatial distribution of street types in downtown Guiyang (see Figure 3).

The street spatial patterns in downtown Guiyang are concluded as follows: compact (540) > closed (483) > open (95) > semi-open (29); in terms of distribution, the closed street spaces are located in the city center, while the closed streets are surrounded by compact streets. The two types of streets are in typical commercial, office, and living areas and most of them have two narrow lanes for two-way traffic. The semi-open and open street spaces are located close to hills, squares, large public buildings, and express roads. Such a distribution structure is closely related to the type of urban street vitality and the degree of street vitality.

### 4.2. Street Vitality Classification and Leveling Results 

**Figure 4:**
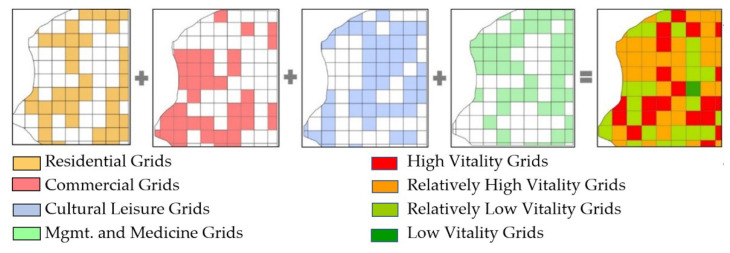
Identification of the type and level of grid vitality based on POI data.

As shown in Figure 4, the layout of street vitality types is cultural leisure vitality (47 grids), accounting for 54.02% of the total; management and medicine (46), 52.87%; and residential and commercial are both 40, accounting for 45.98%, respectively. In terms of the layout features of the street vitality types, streets that boast residential and management and medicine features are more evenly distributed, while commercial vitality is more concentrated in the west and south-west of the study area, and cultural leisure vitality is more seen in the center and north-east regions.

The street vitalities can also be classified according to their levels. The percentage of the grids considered to have high vitality levels is 25.29%; it is 44.83% with relatively high vitality, 28.74% with relatively low vitality, and 1.15% with low vitality levels. An investigation of space distribution of vitality levels shows that most of the study area is covered by grids with high and relatively high vitality levels that stretch out in patches.

### 4.3. Vitality Types and Levels in Different Street Spatial Patterns

**Figure 5:**
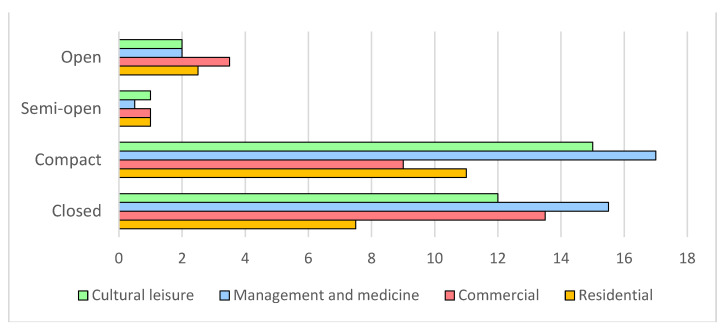
Layout of vitality types against street space patterns.

As shown in Figure 5, commercial vitality occupies the largest share in open street spaces at 35%, followed by lifestyle, which stands at 25.00%. In semi-open street spaces, the four vitality types are equally mixed with residential, commercial, and management and medicine accounting for 28.57%, respectively, and cultural leisure accounting for 14.29%, which is relatively lower. It is noteworthy of our attention that in compact and closed street spaces, the most widely distributed vitality type is cultural leisure, with 32.69% and 31.96%, respectively, whereas in compact spaces, others (28.85%) outperform those of closed spaces (24.74%). The same pattern is observed for the residential category, which accounts for 21.15% of vitality in compact spaces and 15.46% in closed spaces. The proportion of commercial vitality is higher in closed street patterns (27.83%) than in compact street patterns (17.31%).

**Figure 6:**
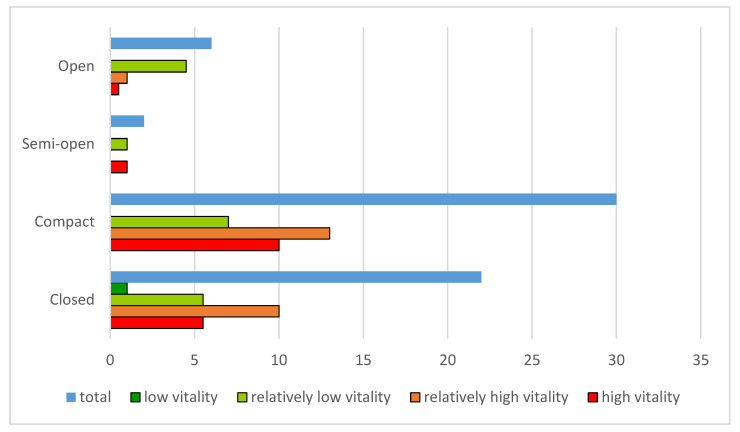
Layout of vitality levels against different street space patterns.

In general, compact street spaces enjoy the highest level of vitality. Among compact streets, 33.33% are high vitality streets and 43.33% are relatively high vitality streets. Closed street patterns are the second highest, with high vitality accounting for 25.00% and relatively high vitality accounting for 45.45%; in open street spatial patterns, the vitality level is the lowest, with 75.00% of the streets considered to be relatively low vitality streets (see Figure 6).

## 5. Discussions

### Effectiveness and Universality of Research Method and Data Processing

This paper proves that the space data in SVI are feasible for the quantified classification of urban street spaces, avoiding uncertainty in spatial pattern classification. Meanwhile, the POI data help to accurately identify street vitality types and layout features. The two quantitative methods for street analysis provide a sound foundation for the quest into the relationship between urban street space and vitality. Moreover, a new method and perspective are introduced for urban street research when grids are used to breakdown the spatial layout of street spaces and vitality types.

## 6. Conclusions

Driven by quick urbanization, the street vitality types in the central area streets of Guiyang continue to overlap and the street space form is undergoing rapid transformation. Hence, Guiyang is in urgent need of clarifying the relationship between street space form, street vitality type, and street vitality strength, so as to provide guidance for shaping street space scientifically and efficiently.

### 6.1. Regularity Found in Layout of Vitality Type and Strength in Various Spatial Patterns

In terms of vitality types, an obvious difference is found in Guiyang regarding the layout of different vitality types in different spatial patterns. The commercial vitality has the widest layout in closed street spaces; residence, cultural leisure, and management and medicine are mostly found in compact spaces; and in semi-open street spaces, all vitality types show the lowest distribution.

In terms of vitality strength, closed and compact street spaces are stronger than semi-open and open. Compact street spaces registered as the highest strength level, whereas the semi-open spaces registered as the lowest.

### 6.2. Compact Street Spaces Should Be Prioritized in Designing Street Space Renovation

Among the four street spatial patterns in central Guiyang, compact street spaces show the highest level of vitality and most diverse vitality types, also making them the best street patterns for dense and rich vitality. As a result, it is recommended that more emphasis should be given to compact street spaces in urban and street design in Guiyang. To be specific, on one hand, this would strengthen the refined design of existing compact street spaces to give full play to the street vitality carried in such street spaces and, on the other, it would intentionally cultivate new compact street spaces in the city to promote urban vitality.

### 6.3. Cultural Leisure Vitality Is Most Adaptive to Street Spatial Patterns

In central Guiyang, it can be found that cultural leisure vitality is the most widely distributed vitality type in its street spaces, especially in compact and closed patterns where cultural leisure outnumbers others extensively. This shows that cultural leisure vitality in central Guiyang streets can adapt to different spatial patterns and co-exist with them, which is highly conducive to both the mental and physical health of the Guiyang people [51].

## Figures and Tables

**Table 1:** Spatial definition and importance of indicators.

No.	Category	Spatial Definition and Importance
1	Street Width	Distance between the edges of the street view. The width is the field of view for a user.
2	Street Length	Length of the centerline of a block. Street length is the depth of the street view for a user.
3	Total Building Numbers	Total number of buildings along the street. It reflects the granularity of the street view.
4	Building Height	Average building height along the street. It is an important indicator of the street enclosing degree and determines the user’s range of vision.
5	Building Cross-Section Ratio	Average building height to width ratio. It describes the interaction of these dimensions.
6	Street Wall Continuity	Ratio of street frontage building width to street length. It describes the interaction between street buildings.
7	Ratio of Total Building Numbers to Street Length	The count of buildings along both sides of a segment standardized by centerline length. It reflects the density of the block.
8	Height Variance	The difference between the tallest building and the lowest building along the street. It describes the continuity of street enclosing degree.

Source: Harvey Chester Wollaeger, 2014 [48].

**Table 2:** Quantitative Street Space Indicators.

No.	Category	Sub-Category	
1	Street Width	Street Width	
2	Street Length	Street Centerline Length	
3	Total Building Numbers	Total Building Numbers	
4	Building Height	Average Building Height	Higher Side
			Lower Side
5	Building Cross-Section Ratio	Average Building Height to Width Ratio	Higher Side Ratio
			Lower Side Ratio
6	Street Wall Continuity	Ratio of Street Frontage Building Width to Street Length	More Continuous Side
			Less Continuous Side
7	Ratio of Total Building Numbers to Street Length	Ratio of Total Building Numbers to Street Length	
8	Height Variance	Difference between High End and Low End of the Building	

Source: Harvey Chester Wollaeger, 2014 [48].

**Table 3:** Street space indicators of core area in downtown Guiyang.

Building Numbers		Street Centerline Length (m)	Street Width (m)	Building Height (m) (Lower Side)	Building Height (m) (Higher Side)	Cross-Section Ratio 1	Cross-Section Ratio 2	Street Wall Continuity 1	Street Wall Continuity 2	Building Numbers/ Street Length	Height Variance (m)
7398	SD	148.75	12.84	10.32	19.7	0.66	1.26	0.42	0.59	0.12	7.77
	Mean	121.10	9.81	9.93	18.74	5.59	9.16	1.83	0.32	0.97	8.55
	CV (%)	122.83	130.88	139.27	105.12	11.80	13.80	22.90	184.43	12.37	90.87

Source: Authors’ calculations.

**Table 4:** Street Spatial Component Matrix.

	1	2	3	4
Building Numbers	0.372	0.650	0.428	0.110
Street Width	0.362	0.191	−0.459	−0.026
Building Height (Lower Side)	0.728	0.158	−0.050	−0.410
Building Height (Higher Side)	0.810	−0.265	−0.116	0.073
Street Centerline Length	0.152	0.549	0.594	0.224
Cross-Section Ratio 1	0.555	−0.627	0.310	0.058
Cross-Section Ratio 2	0.531	−0.697	0.279	0.185
Street Wall Continuity 1	0.662	0.336	−0.056	−0.348
Street Wall Continuity 2	0.670	0.105	−0.009	0.533
Building Numbers Street Length	0.110	0.125	−0.410	0.566
Height Variance	0.594	0.266	−0.297	0.189

Source: Authors’ calculations.

**Table 5:** Classification of street space in downtown Guiyang.

No.	Type	Number of Streets (Sections)	Building Numbers	Height Range (m)	Street Wall Continuity
1	Closed	593	8	10.80–24.08	0.59–0.71
2	Compact	34	10	8.98–15.32	0.57–0.71
3	Semi-open	459	6	8.98–18.9	0.33–0.54
4	Open	64	6	7.35–13.24	0.31–0.43

Source: Authors’ calculations.

**Table 6:** Street functions based on Baidu POI.

Street Vitality Category	Baidu POI Category
Residencial	Residential buildings, utility services
Commercial	Enterprises, shopping, catering, and accommodation services
Cultural leisure	Science, education, culture, sports, leisure, and scenic beauty
Management and medicine	Government and health care

Source: Collated by the author.

## Data Availability

All data used in this study are presented in the manuscript.
